# The Challenge of Dimethyl Fumarate Repurposing in Eye Pathologies

**DOI:** 10.3390/cells11244061

**Published:** 2022-12-15

**Authors:** Federico Manai, Stefano Govoni, Marialaura Amadio

**Affiliations:** 1 Department of Biology and Biotechnology “L. Spallanzani”, University of Pavia, 27100 Pavia, Italy; 2 Department of Drug Sciences, Section of Pharmacology, University of Pavia, 27100 Pavia, Italy

**Keywords:** dimethyl fumarate, DMF, Nrf2, Keap1, eye, retina, age-related macular degeneration, AMD, inflammation, repurposing, ocular disorders

## Abstract

Dimethyl fumarate (DMF) is a small molecule currently approved and used in the treatment of psoriasis and multiple sclerosis due to its immuno-modulatory, anti-inflammatory, and antioxidant properties. As an Nrf2 activator through Keap1 protein inhibition, DMF unveils a potential therapeutical use that is much broader than expected so far. In this comprehensive review we discuss the state-of-art and future perspectives regarding the potential repositioning of this molecule in the panorama of eye pathologies, including Age-related Macular Degeneration (AMD). The DMF’s mechanism of action, an extensive analysis of the *in vitro* and *in vivo* evidence of its beneficial effects, together with a search of the current clinical trials, are here reported. Altogether, this evidence gives an overview of the new potential applications of this molecule in the context of ophthalmological diseases characterized by inflammation and oxidative stress, with a special focus on AMD, for which our gene–disease (*KEAP1*-AMD) database search, followed by a protein–protein interaction analysis, further supports the rationale of DMF use. The necessity to find a topical route of DMF administration to the eye is also discussed. In conclusion, the challenge of DMF repurposing in eye pathologies is feasible and worth scientific attention and well-focused research efforts.

## 1. Dimethyl Fumarate: Chemical Properties and Mechanisms of Action

Dimethyl fumarate (DMF; IUPAC name: dimethyl (*E*)-but-2enedioate) is a methyl ester of fumaric acid (FA) belonging to the family of fumaric acid esters (FAEs), molecules identified in nature in the *Fumaria officinalis*. This small molecule (MW: 144.13 g/mol) is an α,β-unsaturated carboxylic ester derived through the reaction of FA with methanol in the presence of sulfuric acid [1]. DMF and its active metabolite monomethyl fumarate (MMF) are compounds that exhibit a plethora of biological effects, ranging from anti-inflammatory and immunomodulatory properties to radio-sensitizing activities [2,3,4,5,6,7,8]. Oral administration of DMF leads to its rapidly hydrolyzation by the esterases present in the gastrointestinal (GI) tract, thus leading to the formation of MMF, whose half-life is 36 h. Systemic levels of MMF reach the peak after 2–2.5 h; conversely, DMF is not found in circulation [9,10].

The central mechanism of action of DMF is represented by the activation at low concentrations of the nuclear factor erythroid 2 [NF-E2]-related factor 2 (Nrf2, Nfe2l2) and its subsequent translocation in the nucleus. Specifically, the induction of this pathway is mediated through the reaction of DMF with Kelch-like ECH-associated protein 1 (Keap1), a cytoplasmic repressor of Nrf2. At basal condition, Keap1 forms an inactive complex with Nrf2, promoting its ubiquitination and proteasomal degradation through its E3 ubiquitin ligase activity [11]. As a cysteine-rich protein, Keap1 is one of the main targets of the reaction promoted by DMF (i.e., succination), which leads to the Keap1 conformational change responsible for Nrf2 release [12,13]. The consequent translocation of Nrf2 into the nucleus, leads to the binding of this transcriptional factor to antioxidants response elements (ARE) in the genome, thus promoting the expression of a variety of antioxidant and detoxifying genes, such as *Heme Oxygenase-1* (*HO-1*), *NAD(P)H Quinone Dehydrogenase 1* (*HQO1*), and *γ-Glutamylcysteine Synthetase* (*GCLC*) [14,15,16]. Conversely, at a high concentration, DMF induces apoptosis in different cell lines [17].

Notably, increasing interest has raised in the last years regarding the crosstalk between Nrf2 and autophagy. Specifically, the autophagy adaptor protein SQSTM1/p62 can promote the autophagy degradation of Keap1, thus inducing promoting the nuclear translocation of Nrf2 [18]. It has been suggested that the SQSTM1/p62-mediated degradation of Keap1 may be also promoted by sestrins and/or mTORC1 [19,20]. Furthermore, Nrf2 can induce the transcription of *TP53*, which is a well-known autophagy inducer in the presence of DNA damage [21,22]. Furthermore, a positive feedback loop was described, since Nrf2 can induce autophagy upon oxidative stress responses [23]. This finely regulated crosstalk can also explain the DMF-mediated autophagy induction observed in microglial cells [24].

According to the tissue and the cellular context, DMF is also able to induce a variety of cellular responses in a Nrf2-independent mechanisms. Particularly, it has been demonstrated that DMF can perturb cellular metabolism leading to different outcomes: for example, succination and consequent inactivation of glyceraldehyde 3-phosphate dehydrogenase (GAPDH) inhibit aerobic glycolysis in myeloid and lymphoid cells, thus mediating anti-inflammatory effects [25]. A metabolic crisis in pancreatic carcinoma cells was described after DMF treatment through the down-regulation of methylenetetrahydrofolate dehydrogenase (MTHFD1) and methylenetetrahydrofolate dehydrogenase (NADP+ Dependent) 1 Like (MTHFD1L), two enzymes implicated in folate metabolism [26]. The anti-inflammatory properties of DMF are also exerted through the inhibition of the nuclear translocation of both the canonical and non-canonical nuclear factor kappa-light-chain-enhancer of activated B cells (NF-κB) protein [27,28]. This inhibition seems to be mediated by direct interaction and modulation of hydroxycarboxylic acid receptor 2 (HCAR2) [29]. Furthermore, both the NF-κB and Erk1/2 pathway can be down-regulated by DMF treatment through the inhibition of K63 and M1 polyubiquitin chain formation, which is essential to link Toll-like receptor (TLR) and to its down-stream effectors (i.e., NF-κB and Erk1/2) [30]. Another target of DMF is the hypoxia-inducible factor-1α (HIF-1α), leading to the disruption of its complex with the heat shock protein 90 (Hsp90) and affecting its folding and maturation [31]. DMF can also modulate glutathione metabolism in both Nrf2-dependent and independent ways [32,33,34]. Notably, as described by proteomic analysis, other essential cellular responses are mediated by the reaction of succination involving enzymes like thioredoxin-1 (TRX1), inhibitor of κβ kinase (IKKβ), and protein kinase C Ө (PKCӨ) [35,36].

## 2. Current Clinical Applications and Clinical Trials

Considering the role of Nrf2 in the oxidative stress response and aging [37,38], neurodegenerative disorders [39], and inflammation [40], this pathway became a candidate target in many diseases and DMF a candidate drug. Nowadays, several electrophilic and non-electrophilic Nrf2 activators are under investigation in clinical trials to assess their therapeutic efficacy in different pathological contexts [41]. Besides DMF, other compounds, such as cyanidin-3-*O*-glucoside (C3G), have been studied in the last decade for their capability to induce the Nrf2 pathway [42,43,44,45].

DMF/MMF, as well as other fumaric acid esters (FAEs), are molecules of great clinical interest. Specifically, DMF is a relatively well-tolerated molecule whose mechanisms of action are well characterized [46,47,48,49]. The therapeutic efficacy of DMF is primarily mediated by the activation of the Nrf2 pathway [12,13] and by the modulation of HCAR2 [29]. Nowadays, approved indications for DMF include psoriasis and multiple sclerosis (MS).

### 2.1. Psoriasis

Psoriasis (*Psoriasis vulgaris*, PV) is a chronic inflammatory disorder of the derma, with a mean frequency of ~2% worldwide, affecting men and women equally. The disease, whose onset is generally between 15 and 30 years of age, is characterized by variable expressivity, in which symptoms may be mild or severe [50]. The etiology of psoriasis is determined by both genetics and environmental factors. Genome-wide association studies (GWASs) identified several genes (more than 60) that are generally involved in T-helper 17 (Th17) cells activation [51]. Patients show red and squamous/flaky patches on the skin usually localized on the scalp, lower back, and joints. These lesions are caused by abnormal proliferation of keratinocytes and infiltration of immune cells: specifically, dendritic cells (DCs) and T-cells in the dermis and neutrophils in the epidermis [52].

As reported in literature, the skin lesions of psoriatic patients show increased DCs, which induce T-cells as well as the production of Th1 cytokines, Tumor Necrosis Factor-α (TNF-α), and inducible nitric oxide synthase (iNOS) [53,54]. Additionally, Th17 cells are involved in psoriasis pathogenesis, through the release of some interleukins, such as interleukin-17 (IL-17) and IL-22 [55].

DMF systemic treatment was identified as a potential therapy for psoriasis according to previous evidence collected on FA treatment of psoriatic patients in the late 1950s. DMF, in combination with monoethyl fumarate salts, was first licensed for psoriasis treatment in 1994 in Germany with the commercial name of Fumaderm^®^ [56]. *In vitro* and *in vivo* studies disclosed the beneficial molecular actions exerted by DMF in the context of psoriasis. Systemic DMF treatment leads to a decrease in IL-6, IL-17, IL-22, granulocyte-macrophage colony-stimulating factor (GM-CSF), transforming growth factor-α (TGF-α), and interferon-γ (IFN-γ) and, conversely, induces an increase in the levels of IL-10. Furthermore, DMF enhances the activation of T regulatory (Treg) cells and inhibits Th17 cells [57,58]. Notably, DMF’s beneficial effect in psoriasis is mediated by the depletion of glutathione-*S*-transferase [33] and by the reduction in the pro-inflammatory neutrophil population [59].

Recent *in vivo* findings demonstrated that the Nrf2-dependent mechanism of action of DMF plays an important role in psoriasis treatment. Specifically, DMF administration to *Nrf2^+/+^* mice model of psoriasis leads to attenuation of the symptoms and rescues epidermal differentiation; conversely, no effects are detectable in *Nrf2^−/−^* mice models [60].

### 2.2. Multiple Sclerosis

Multiple sclerosis (MS) is a chronic immune-mediated disorder which affects the central nervous systems (CNS), leading to demyelination and consequent neuronal degeneration. MS primarily affects young adults, predominantly women. The etiology is mediated by both genetic and environmental factors [61]. The majority of patients (~85%) present the relapsing-remitting form of the disease (RRMS), whereas the remaining patients (~15%) show a continue progression of the disorder, defined as primary progressive MS (PPMS) [62]. The principal histopathological hallmark of MS is the presence of focal lesions in grey and white matter, characterized by neuronal demyelination and astrocytic scars. Despite the original dogma, the underlying inflammatory process is mediated by both T- and B-lymphocytes. Specifically, T-cells belong to the Human Leukocyte Antigen class I (HLA-I) CD8^+^ class whereas B-cells show either the CD20^+^ or plasma cell phenotype, according to the stage of the disorder. Generally, immune cells in MS patients invade the perivascular space and the parenchyma [63].

MS therapeutic strategies are mainly based on inhibiting neuroinflammation or promote neuroprotection and remyelination. DMF exerts beneficial effects on both inflammation and neuroprotection, thus becoming an excellent reference compound in the treatment of MS. Particularly, different clinical trials showed how DMF is well-tolerated by MS patients at different doses, leading to a remission of the symptoms or to inhibition of relapses in RRMS [64,65,66,67].

DMF modulates both microglia/astrocytes and immune cells. The anti-inflammatory effect on glial cells is due to the inhibition of the release of several pro-inflammatory molecules, i.e., nitric oxide (NO), IL-1β, IL-6, and TNF-α [68]. The DMF-induced modulation of microglia is also mediated by the HCAR2 signaling pathway as well as by Nrf2, thus promoting a shift of the glial phenotype from a pro-inflammatory to a non-activated one [6,69]. Moreover, the activation of Nrf2 pathway leads to a reduction in the inflammatory levels in astrocyte through glutathione and HO-1; overall, the Nrf2-mediated beneficial effects of DMF were also demonstrated *in vivo*: neuroprotection after DMF treatment was observed in a model of hypoxic-ischemic damage [70,71,72]. Recently, it has been demonstrated that astrocyte protection against oxidative stress is mediated by the Nrf2-induced up-regulation of oxidative stress-induced growth inhibitor 1 (OSGIN-1), a protein implicated in the oxidative stress response [73]. Notably, DMF effects on the immune system play a key role in MS treatment. DMF can induce immunosuppression through HO-1 induction in presence of glutathione depletion [34]. Recently, it has been demonstrated that DMF can alter the phenotypic profile of B- and T-cells, thus inhibiting their activation in MS patients and promoting the release of beneficial cytokines, such as IL-4 [74,75]. Another described effect of DMF in MS patients is the inhibition of Th1/Th17 cell differentiation and the reduced expression of circulatory pro-inflammatory cytokines [76].

### 2.3. Ongoing Clinical Trials and New Potential Indications

To date, several clinical trials are ongoing to study the effects of DMF in the contexts of different disorders. The majority of these studies are focused on MS (95) and psoriasis (12). In Table 1, all the clinical trials involving DMF/MMF in different disorders are reported, excluding the well-known applications in psoriasis and MS (all the studies involving patients with a diagnosis of MS or psoriasis were excluded).

Beneficial effects of DMF in autoimmune disorder have been widely described in the literature: an example is represented by the therapeutic effect of FAEs in the treatment of discoid lupus erythematosus [77]. Pre-clinical studies demonstrated that osteoclasts modulation by DMF plays a key role in rheumatoid arthritis (RA): particularly, DMF leads to a decrease in receptor activator of nuclear factor kappa-B ligand (RANKL) levels after ROS generation through Nrf2 activation. Moreover, DMF can alter osteoclasts differentiation by inhibiting nuclear factor of activated T-cell 1 (NFATc1) and activating the Erk1/2 and p38 MAPK pathways [78,79]. Modulation of the immune system exerted by DMF also plays an important role in the treatment of systemic sclerosis [80]. The beneficial effects of DMF were also demonstrated in cancer. Specifically, this molecule can induce cell death in different types of blood cancers [81,82,83]. Similarly, *in vitro* and *in vivo* pre-clinical studies demonstrated that in solid tumors DMF exerts a therapeutic potential both alone and in combination with other compounds [26,84,85,86,87].

The increasing number of clinical trials in cerebrovascular diseases aimed at studying the therapeutic potential of DMF is based on the increasing *in vitro* and *in vivo* pre-clinical evidence that has been raised in the last years [88]. Considering the growing and its anti-inflammatory and antioxidant properties, DMF has the potential to be considered/used more broadly in other pathologies characterized by inflammation and/or oxidative stress.

## 3. DMF and Eye Disorders: State of the Art

The Nrf2-dependent mechanism of action of DMF confers attractiveness from a therapeutic point of view to this molecule in the context of ocular diseases [89]. Indeed, impairment or loss of Nrf2 content or activity were described in several ocular pathologies, such as uveitis, diabetic retinopathy, retinal ganglion cells (RGCs) degeneration, AMD, cataracts, and glaucoma [90,91,92,93,94,95]. *Nrf2^−/−^* animal models display similar characteristics reminiscent of AMD. The knockout (KO) of *Nrf2* also leads to an increase of RGCs death compared to wild-type (WT) mice in a model of oxidative stress-mediated RGCs degeneration as well as an increase in intercellular adhesion molecule 1 (ICAM-1), IL-6, TNF-α, cyclooxygenase 2 (COX-2), iNOS, and monocyte chemoattractant protein 1 (MCP-1) compared to WT mice after the administration of lipopolysaccharide (LPS). Interestingly, administration of Nrf2 inducers can revert these phenotypes, thus mitigating the cell death/degeneration rate and the inflammatory/innate immune response. Genetic studies have also revealed that a single nucleotide polymorphism (SNP) in *NFE2L2* gene is associated with an increased risk for AMD. Furthermore, Nrf2 pathway plays a fundamental role in the maintenance of cellular redox homeostasis during aging, thus confirming its involvement in age-related ocular diseases [96].

**Figure 1:**
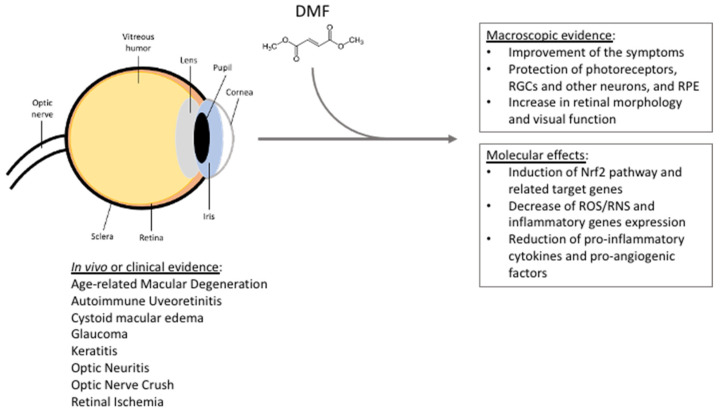
Summarizing scheme reporting DMF beneficial effects in the context of eye pathologies, based on *in vivo* studies, clinical trials, and clinical and case studies. See the text for further details. RGCs = retinal ganglion cells; RPE = retinal pigment epithelium; ROS/RNS = reactive oxygen/nitrogen species.

Increasing evidence suggested that DMF has a strong potential in eye pathologies and might be used in several ophthalmological contexts. This hypothesis is based on the evidence collected on MS patients. Optic neuritis is the first demyelinating symptom that occurs in 15–20% of MS patients and almost 50% of patients experience this problem during the disease progression [37]. *In vivo* studies have also revealed that DMF, as well as other canonical treatment of MS, exerts a higher beneficial effect than IFN-γ on RGCs by preventing their loss in MS patients [97]. Moreover, two case reports described the beneficial effects of systemic DMF administration in counteracting the symptoms of uveitis and cystoid macular edema [98,99]. Based on this evidence, a clinical trial started in 2022 with the aim to study the effects of oral administration of DMF in AMD patients. Specifically, 30 patients between 55- to 85-years-old with central or non-central geographic atrophy (GA) in at least one eye, and visual acuity between 25/25 and 20/200 in the affected eye, will be treated with 120 mg DMF twice a day for the first week, and then with 240 mg DMF twice a day for 51 weeks (NCT04292080). The results will be compared with those collected in a cohort of AMD subjects under no specific treatment. Table 2 reports an exhaustive list of the literature evidence and relative references supporting the DMF potential use in ophthalmology. Figure 1 summarizes the main DMF beneficial effects referring to *in vivo* studies and evidence in human subjects in the context of eye pathologies.

*In vitro* studies have elucidated the protective role of DMF on retinal pigment epithelial cells (RPE), essential for photoreceptor homeostasis and participating to the constitution of the retinal–blood barrier (RBB). Specifically, DMF can induce an increase in GSH in RPE cells [111] and plays a key role in RPE cells response to several harmful stimuli. DMF inhibits apoptosis in model of high-glucose (HG) stress based on ARPE-19. Moreover, DMF treatment leads to a decrease in iNOS, COX-2, IL-1β, and vascular endothelial growth factor (VEGF) [115]. DMF-mediated anti-inflammatory effects were also described in primary human RPE cells treated with TNF-α for 5 days. Again, pre-treatment with DMF restored the normal cellular phenotype, promoting a reduction in IL-6, IL-8, MCP-1, and mitochondrial oxidative phosphorylation system (OXPHOS) protein levels. These modifications were also associated with a rescue of normal mitochondrial morphology as well as a metabolic normalization, through the regulation of 6-phosphofructo-2-kinase/fructose-2,6-bisphosphatase 3 (PFKFB3) and pyruvate kinase isozyme M2 (PKM2) [117]. The protective effects of DMF through the activation of Nrf2 and down-stream effectors (e.g., HO-1 and p62) was also demonstrated in ARPE-19 cells exposed to different toxic stimuli, such as oxidative stress, lipid peroxidation products, and protein misfolding [116]; in particular, under pro-oxidant stress, DMF was able to both counteract the cell mortality and decrease ROS levels.

Notably, MMF plays a protective role, even if in other cells of the eye. This molecule can regulate the proton-coupled folate transporter (PCFT) in Müller cells, thus preventing reactive gliosis, and can induce the sodium-independent cysteine/glutamate antiporter solute carrier family 7 member 11 (SCL7A11) in RPE cells, thus potentiating its antioxidant signaling [101,112].

All the evidence was corroborated in *in vivo* studies. Treatment with DMF of BALB/c mice infected with *Herpes simplex* virus 1 (HSV-1, KOS strain) showed a reduction in the onset of severe keratitis and a consistent decrease of CD3^+^, CD11b^+^, GR-1^+^, and F4-80^+^ cells [100]. Positive results were also collected after the treatment of patients with noninfectious uveitis with FAEs [98].

Other beneficial effects were described in the context of optic nerve injury. Particularly, treatment with increasing concentrations of DMF was able to ameliorate the outcome of C57BL/6J mice subjected to optic nerve crush (ONC). An increase in Tubβ3^+^ RGCs and in the amplitude of positive scotopic threshold response (pSTR) was detected, together with an increase in Nrf2 and HO-1 protein levels [107]. This evidence, together with the evidence collected by You and colleagues [97], suggests a potential application of DMF in the context of glaucoma. In another study, DMF was administered using a different therapeutic scheme in two mouse models based on light-induced photoreceptor loss and ONC, respectively. In this case, DMF exerted a beneficial effect through the reduction of reactive microglia and the increase in GSH levels. Conversely, no effects were detected on ONC [108]. The same beneficial effects were observed in a model of optic neuritis. This pathology occurs frequently in RRMS patients, which experience acute monocular vision loss. Additionally, in *in vivo* animal models based on the inoculation of myelin oligodendrocyte glycoprotein peptide (i.e., MOG_35–55_) in C57BL/6J mice, the DMF administration led to a decrease in the severity of the inflammation thanks to an increase in Nrf2 levels [106]. The immunomodulatory action of DMF was also demonstrated in rat models of endogenous uveitis and corneal lymphangiogenesis. In the former study it was observed that DMF impaired NO, TNF-α, CD68, CD20, CD25, and CD8 levels; the latter showed that DMF treatment induced a reduction in MCP-1, TNF-α, IL-6, IL-1β, VEGF, and macrophages infiltration through NF-κB inhibition [109,110].

Other studies strengthened the rationale of the use of DMF in the context of ocular diseases. Specifically, ocular disorders represent a common comorbidity in vascular diseases, such as diabetes. An example is diabetic retinopathy (DR), a pathological condition characterized by vascular and endothelial dysfunctions [119]. Nrf2 dysfunction is strongly associated with vascular-related eye pathologies, as reported in several *in vivo* studies, which demonstrates a reduced activation of Nrf2-signaling pathway in DR and low circulatory levels of this protein [92,120,121]. Considering these premises, DMF efficacy was also investigated in *in vitro* models of endothelial cells, such as HUVEC and HBMVEC [113,114]. These two studies showed that DMF was able to reduce the TNF-α-mediated pro-inflammatory response in HUVEC, but no effects were detected in HBMVEC. Notably, it has been recently demonstrated that DMF treatment is able to activate Nrf2/HO-1 pathway and to reduce basal ROS levels in human primary retinal endothelial cell (HREC) line, and to protect these cells from the damage of HG stress [118], thus further highlighting the beneficial effects of DMF.

The efficacy of DMF in ocular pathologies was also demonstrated by a case report of Kofler and colleagues [99]. Oral therapy with Fumaderm^®^ was applied to an 88-year old woman affected by cystoid macular edema. The oral administration was initially 40 mg/die and then was increased to 480 mg/die. Since the patient experienced abdominal pain and diarrhea, DMF administration was reduced to 240 mg/die. After 24 months the patient showed a progressive improvement of her condition: after nine months a reduction in the number of cysts was observed; a decrease in retinal thickness was detected after 12 months until the symptoms regressed from severe to mild.

Additionally, MMF promoted similar beneficial effects. C57BL/6J *Nrf2*^−/−^ mice models of ischemia/reperfusion damage showed an improvement of the eye’s health. After 48 h post-treatment, authors described an increase in Nrf2 levels and in retinal function (measured by electroretinography, ERG) and a decrease in inflammatory gene expression, in gliosis level, and neuronal loss in the ganglion cell layer (GCL) [105]. An MMF-mediated increase in HO-1 and a concomitant decrease in caspase-1, IL-1β, TNF-α, and in NLR family pyrin domain containing 3 (NLRP3) was also described in albino BABL/c mice exposed to bright white light for one hour to mimic light-induced retinopathy [104]. In *in vitro* and *in vivo* models of sickle cell disease (SCD), the treatment with MMF was able to induce γ-globin and fetal hemoglobin and to reduce the levels of oxidative stress and inflammatory markers [102,103].

## 4. DMF and Eye Disorders: Rationale and Comparison with Other Molecules

Several molecules have been identified as inducers of the Nrf2-pathway and some of them are already under investigation in clinical trials. As described [41,122], many Nrf2 activators showed promising therapeutic potential in various types of disorders. However, as reported, the application of some of these compounds in clinical practice may be limited by a variety of factors. For example, Michael acceptors, such as olefins, are inhibitors of the Keap1-Nrf2 binding, thus leading to the release and consequent activation of Nrf2; however, despite their therapeutic efficacy, these molecules show a classical dose-response curve in which at low concentrations they react with the desired targets, such as Keap1, whereas at higher concentrations they induce cytotoxicity due to off-target effects derived by their chemical reactivity. Another example is represented by natural compounds, such as curcumin and related agents. These molecules share a similar mechanism of action with Michael acceptors and received great attention due to their biological action. Unfortunately, the use of these molecules is limited by their instability, poor or not-evaluated *in vivo* bioavailability, and rapid degradation.

Different natural compounds were investigated in the context of eye pathologies. An example is sulforaphane (SFN), a molecule present in cruciferous vegetables (*Brassicaceae* family). Both *in vitro* and *in vivo* studies demonstrated that this compound played Nrf2-mediated beneficial effects against photooxidative damage in RPE cells and mitigated light-induced retinal damage in mice [123,124]. SFN, as already described for DMF, can protect eye cells (e.g., photoreceptors, retinal pigment epithelial cells, lens cells, retinal endothelial cells, and retinal pericytes), and it exerts anti-inflammatory effects preventing microglia activation [125,126,127,128,129,130]. Despite this evidence, SFN is not suitable for clinical practice due to some limitation. Particularly, SFN treatment *in vivo* highlighted the presence of unsolved challenges in its clinical applications (e.g., source selection, extraction, and not characterized protocols) [131,132,133,134]. Nowadays, no clinical trials involving SFN in eye pathologies are available.

Same considerations can be made for other natural compounds, such as flavonoids, carotenoids, and vitamins. All these compounds showed beneficial effects *in vitro* and *in vivo* models of ocular pathologies. However, issues regarding the source, bioavailability in humans, and the lack of well-designed clinical trials limit their application in clinical practice [135].

Novel interesting approaches might be represented by molecular hybrids with an Nrf2-dependent mechanism of action like that characterizing DMF. An example is represented by natural-inspired hybrids (NIHs), a family of compounds that were synthesized starting from the chemical structure of curcumin [116]. This strategy seems to be encouraging to overcome the issues that characterized the use of natural compounds, like curcumin (e.g., short half-life, low solubility, and rapid metabolism). These molecules can modulate the Nrf2 and NF-κB pathways in different cell lines, such as SH-SY5Y and THP-1 [136,137]. The efficacy of NIHs was also studied in the context of the eye. Particularly, in the last two years their chemical properties as well as their beneficial effects *in vitro* and *in vivo* model of ocular pathologies have been elucidated. In ARPE-19 cells the treatment with these compounds leads to an increase in Nrf2 protein levels, as well as in its down-stream effectors HO-1 and p62. These effects promoted cytoprotection against different toxic stimuli, such as oxidative stress and dysfunction in protein homeostasis [116,138]. Notably, the NIH lead compound was able to increase Nrf2 levels in *ex vivo* retinal explants, promoting its nuclear translocation and the induction of down-stream effectors, such as HO-1 and NQO1 [139]. Moreover, NIH treatment promoted a reduction in caspase-1 and GFAP levels in presence/absence of oxidative stress, thus underlying its ability to counteract reactive gliosis and promote retinal cell viability.

## 5. AMD: A Paradigm for DMF Repurposing

Among ocular diseases, AMD may represent an interesting indication for DMF therapeutic applications. This neurodegenerative disorder affects elderly people worldwide and is the major cause of blindness. AMD is classified in dry (~90%) and wet (~10%) forms [140]. The hallmarks in AMD onset consist of RPE degeneration, the increase of ROS levels, and autophagy’s loss of homeostasis [141,142]. The Keap1/Nrf2 pathway plays a key role in the maintenance of RPE cellular homeostasis, as well as in ROS neutralization [143,144]. The importance of this pathway in AMD onset was also demonstrated through the generation of double knockout (dKO) mice for *Nrf2* and *Ppargc1a* [145]. Notably, the loss of these genes led to an age-dependent RPE degeneration, accumulation of ROS, alteration in photoreceptor morphology, and visual impairment. Moreover, a single nucleotide polymorphism (SNP) in the *NFE2L2* gene (rs6726395) has been identified, thus strengthening the association of Nrf2 pathway and AMD [146].

**Figure 2:**
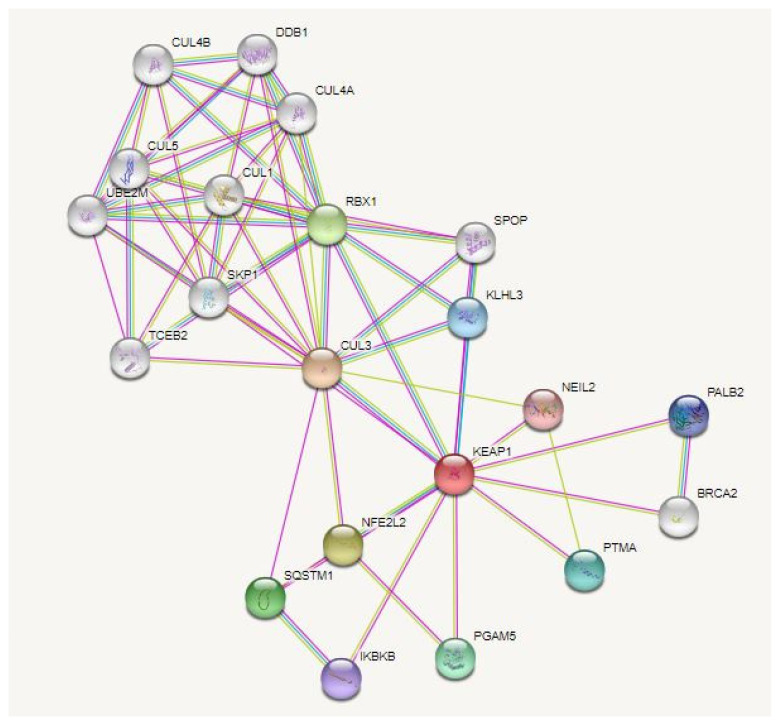
PPI (protein–protein interaction) network of Keap1. In DisGeNet (https://disgenet.org/, accessed on 29 September 2022), the association of Keap1 and AMD was verified. The PPI network for Keap1 was then analyzed using the STRING database (https://string-db.org/, accessed in 29 September 2022); the interactions were analyzed in *Homo sapiens*, setting the confidence basis to 0.4 and selecting only the physical interactions.

As said before, DMF reacts with Keap1 protein leading to Nrf2 activation; we thus examined the link between Keap1 and AMD by consulting the gene–disease DisGeNet database, followed by investigation of Keap1’s multiple physical associations through protein–protein interaction (PPI) analyses (Figure 2); this search revealed that DMF may exert other additional beneficial biological effects on RPE cells than the ones already mentioned.

Specifically, it is possible to identify an interaction between Keap1 and the enzyme NEIL2 (Nei-like 2) that, in mammalian cells, contributes to the UV-induced oxidative stress response together with YB-1 (Y-Box-binding 1) protein [147]. Another interesting interaction is that occurring with BRCA2 (*Br*east *Ca*ncer gene 2) and PALB2 (Partner and Localizer of BRCA2), two components of the DNA repair system mediated by homologous recombination (HR) [148]. Notably, one interactor of Keap1 is RBX1 (RING-box protein one), which is itself an interactor of DDB1 and DDB2 (DNA damage binding protein 1 and 2), two proteins involved in the nucleotide and UV-damage excision repair systems [149].

According to our analyses, as well as literature evidence, the crosstalk between Nrf2 and autophagy, together with the direct interaction between Keap1 and SQSTM1/p62, suggests possible anti-inflammatory properties of DMF mediated by autophagy induction. Indeed, the autophagic process contributes to the modulation of inflammation through different mechanisms, two of which are the degradation of inflammasome-related proteins [150], and the neutralization of oxidative stress [151]. Moreover, autophagy also plays a role in epithelial-to-mesenchymal transition (EMT), which contributes to the onset of RPE’s fibrosis observed in AMD [152] by preventing or reverting it [153,154].

Interestingly, and based on the DMF-induced beneficial effects in PR and RPE cells, we suggest that DMF treatment may also represent a therapeutic approach in the Retinitis Pigmentosa (RP), an inherited disease featured by visual loss and dramatically affecting quality of life of young and adult patients. Specifically, it has been demonstrated that MMF administration (50 mg/kg, intraperitoneally every other day, P14-42) to Pde6βrd10/J (rd10) mice, a model of RP, led to a sustained up-regulation of antioxidant genes through Nrf2 pathway activation [155]. However, since these effects in MMF-treated rd10 mice were not accompanied by significative improvements in the retinal morphology and function compared with untreated diseased controls, the authors concluded that, in their experimental conditions, MMF may not be sufficient to delay the catastrophic PR damage characteristic of the rd10 mouse, and suggested that MMF may be able to prevent, but not restore, PR degeneration [155]. Among others, these findings and related observations suggest that MMF, but more pertinently DMF, which boosts a more robust response than its metabolite MMF [156], may take advantage of different times, concentrations, and/or routes of administrations, as discussed in the next paragraph.

## 6. Adverse Events of DMF Systemic Treatment and Its Potential Topical Application

DMF is currently used as an oral treatment in clinical practice and shows effectiveness, tolerability, and good bioavailability. However, adverse effects (AEs) have been described in this route of administration, particularly affecting the GI tract. The most common AEs, principally observed in females, are diarrhea, stomachache, nausea, vomiting, and cramps [157]. It has been demonstrated that DMF crosses the blood–brain barrier [158] and it may be the same for the blood–retinal barrier; however, for the treatment of eye pathologies, a topical administration—if possible and available—might be more preferable than the systemic route for various pharmacokinetic and pharmacodynamic aspects. For example, compared to the oral route, topical administration of a drug usually allows for: using a lower therapeutic dosage, since systemic distribution is reduced or absent; avoiding or minimizing systemic side-effects; treating patients with alterations in ADME (e.g., low gastrointestinal absorption, liver dysfunction, renal failure), which would be otherwise excluded/damaged by the systemic treatment; easily delivering the drug with no or low off-target effects on other organs/tissues.

The potential DMF topical application has been limited by contact dermatitis evidenced almost four decades ago in patients with psoriasis following a cutaneous DMF treatment [159]. However, the DMF-induced cutaneous toxicity may distress anatomical structures that are phenotypically different, or even absent in the eye; in addition, this reaction may be mediated by other inflammatory factors than the ones present at ocular level. Indeed, the eye is characterized by an “immune privilege”, and its reactions may differ from other body districts [160]. Beyond these hypotheses and considerations, relevant efforts in the discovery of novel delivery systems of DMF have been made, with the aim to improve the tolerability of this molecule and/or its biodistribution to selected body districts. For instance, studies on a cutaneous treatment with a sensitization-free DMF prodrug (isosorbide DMF) [161] on DMF-loaded transethosomes [162] and on vitamin-derived nanolipoidal carriers for brain delivery of DMF [163] showed encouraging *in vitro*, *ex vivo*, and preclinical evidence.

Although not specifically designed for the ocular treatment, these observations indicate that nanotechnological delivery systems of DMF will be likely available for topical administration in eye pathologies soon. Of course, it will be also relevant to find the most suitable formulation to deliver DMF specifically to the retinal cells affected by a given pathology; accordingly, administrations by intraocular injection and eye drops represent feasible, although antithetic routes with respective limitations [164]. Considering that various liposomes, nanoparticles, and microspheres are currently useful delivery systems overcoming the limitations of topic ocular drug administration [165,166], more than one of these approaches may represent a feasible strategy for DMF ophthalmic delivery.

Last, but not least, in virtue of the proven benefits of DMF, its topical treatments may have the potential to prevent or counteract more than one eye pathology, as this drug may display either protective or rescue/therapeutic effects; however, additional preclinical studies will be needed to clarify these points, and to better tailor the DMF use in the panorama of ocular diseases.

## 7. Conclusions

DMF is an effective treatment that has been used for almost 60 years in Europe and is approved for psoriasis and RRMS. The rising evidence of the beneficial effects of DMF in *in vitro* and *in vivo* models of eye disorders encourages its repurposing for the treatment of ocular pathologies. The reasons for DMF attractiveness rely on its mechanism of action, which is mainly mediated by Nrf2 activation. This transcriptional factor is a therapeutic target in eye disorders, such as AMD, in virtue of Nrf2-pathway’s role in the pathogenesis of several ocular affections. Notably, Nrf2 reduced activity is associated with the hallmarks of various ocular disorders: oxidative stress, inflammation, and vascular dysfunction.

In conclusion, the challenge of DMF repurposing in eye pathologies has just begun, and it is already worth further scientific attention and research efforts.

## Figures and Tables

**Table 1:** Clinical trials involving DMF/MMF in different diseases. Exclusion criteria: patients involving a diagnosis of MS or psoriasis. The terminated trials were interrupted for low recruitment.

Topic	Status	Conditions	ID
Autoimmune diseases	Completed	Lupus, cutaneous	NCT01352988
	Completed	Rheumatoid arthritis	NCT00810836
	Terminated	Systemic sclerosis Pulmonary hypertension	NCT02981082
Cancer	Unknown ^1^	Cutaneous T-cell lymphoma	NCT02546440
	Completed	Glioblastoma	NCT02337426
	Terminated	Chronic lymphocytic leukemia	NCT02784834
Cardiovascular disorders	Unknown	Diabetes mellitus	NCT01088165
Respiratory tract	Completed	Obstructive apnea	NCT02438137
	Recruiting	Severe acute respiratory syndrome	NCT04381936
Cerebrovascular disorders	Not yet recruiting	Acute ischemic stroke	NCT04890353
	Not yet recruiting	Acute ischemic stroke	NCT04891497
	Recruiting	Acute ischemic stroke	NCT04890366
	Not yet recruiting	Intracerebral hemorrhage	NCT04890379

^1^ The study has passed its completion date and its status has not been verified in the last two years.

**Table 2:** Studies and clinical trials about DMF applications in the context of ophthalmological diseases. The symbol ↑ indicates “increase”, whereas ↓ indicates “decrease”.

Author and Year	Molecule	Type of Study	Model	Described Effects
PIs: Souied and Camelo, 2022	DMF	*Clinical trial*	55- to 85-year-old patients with central or non-central geographic atrophy in at least one eye with AMD	-
Heinz and Heilingenhaus, 2007 [98]	FAEs	*Case report*	Patients with uveitis	Improvement of the symptoms
Kofler et al., 2018 [99]	DMF	*Case report*	88-year-old woman affected by cystoid macular edema	↓ cysts, retinal thickness, edema
You et al., 2021 [97]	DMF	*Clinical study*	Multiple sclerosis patients	↓ loss of RGCs
				
Heiligenhaus et al., 2004 [100]	DMF	*In vivo*	BALB/c mouse infected with HSV-1 (severe keratitis)	↓ CD3, CD11b, GR-1, F4-80 positive cells
Ananth et al., 2013 [101]	MMF	*In vivo/in vitro*	ARPE-19, primary RPE, *Slc5a8^-/-^* and *Gpr109a^-/-^* mouse	↑ SLC7A11, Nrf2, HIF-α, GSH
Promsote et al., 2014 [102]	MMF	*Ex vivo/in vitro*	hRPE, retinal explants	↑ γ-globin, fetal hemoglobin
Promsote et al., 2016 [103]	MMF	*In vivo/in vitro*	Sickle cell disease mouse model; ARPE-19 cells	↑ retinal morphology and barrier function, visual function, Nrf2, HO-1, NQO1, TRX-1; ↓ VEGF, ICAM-1
Jiang et al., 2019 [104]	MMF	*In vivo*	Albino BALB/c mouse with light-induced retinopathy	↓ NLRP3, caspase-1, IL-1β, TNF-α; ↑ HO-1
Cho et al., 2015 [105]	MMF	*In vivo*	C57BL/6 Nrf2^-/-^ mouse (retinal ischemia/reperfusion)	↑ Nrf2, retinal function; ↓ Inflammatory genes, gliosis, neuronal loss in GCL
Zyla et al., 2019 [106]	DMF	*In vivo*	EAE mouse model of optic neuritis	↑ Nrf2; ↓ optic neuritis severity
Mori et al., 2021 [107]	DMF	*In vivo*	C57BL/6J mouse (Optic nerve crush)	↑ Nrf2, HO-1, Tubβ3^+^ RGCs, pSTR
Dietrich et al., 2021 [108]	DMF	*In vivo*	C57BL/6J mouse (Optic nerve crush; light-induced photoreceptor loss)	↑ GSH; ↓ Iba1, loss of photoreceptor
Yu et al., 2021 [109]	DMF	*In vivo*	Rats undergoing corneal transplantation	↓ MCP-1, TNF-α, IL-6, IL-1β, VEGF, macrophages infiltration
Labsi et al., 2021 [110]	DMF	*In vivo*	Female Wistar rat model of autoimmune uveoretinitis	↓ NO, TNF-α, CD-68, CD-20, CD-25, CD-8 positive cells
				
Nelson et al., 1999 [111]	DMF	*In vitro*	hRPE	↑ GSH
Bozard et al., 2012 [112]	MMF	*In vitro*	Müller cells	Regulation of proton-coupled folate transporter
Haarmann et al., 2015 [113]	DMF/MMF	*In vitro*	HBMVEC	No effects
Gerhardt et al., 2015 [114]	DMF	*In vitro*	HUVEC	↓ MCP-1, NF-κB translocation, IκBα degradation, IL-6, CCL-5, GM-CSF, PDGF-β
Maugeri et al., 2021 [115]	DMF	*In vitro*	ARPE-19 cells under high-glucose stress	↓ Bax/Bcl-2 ratio, VEGF, iNOS, COX-2, IL-1β
Catanzaro et al., 2021 [116]	DMF	*In vitro*	ARPE-19 cells treated with H_2_O_2_, 4-HNE, MG132+Bafilomycin A1	↑ Nrf2, HO-1, p62, cell viability; ↓ ROS
Shu et al., 2022 [117]	DMF	*In vitro*	hRPE treated with TNF-α	↓ IL-6, IL-8, MCP-1, *OXPHOS* genes; ↑ glicolysis
Manai et al., 2022 [118]	DMF	*In vitro*	Human retinal endothelial cells	↑ Nrf2, HO-1, cell viability; ↓ ROS

## Data Availability

Not applicable.
